# Vitamin and Mineral Supplementation and Rate of Gain in Beef Heifers I: Effects on Dam Hormonal and Metabolic Status, Fetal Tissue and Organ Mass, and Concentration of Glucose and Fructose in Fetal Fluids at d 83 of Gestation

**DOI:** 10.3390/ani12141757

**Published:** 2022-07-08

**Authors:** Ana Clara B. Menezes, Kacie L. McCarthy, Cierrah J. Kassetas, Friederike Baumgaertner, James D. Kirsch, Sheri T. Dorsam, Tammi L. Neville, Alison K. Ward, Pawel P. Borowicz, Lawrence P. Reynolds, Kevin K. Sedivec, J. Chris Forcherio, Ronald Scott, Joel S. Caton, Carl R. Dahlen

**Affiliations:** 1 Center for Nutrition and Pregnancy, Department of Animal Sciences, North Dakota State University, Fargo, ND 58108, USA; cierrahjk@hotmail.com (C.J.K.); friederike.baumgrtne@ndsu.edu (F.B.); james.kirsch@ndsu.edu (J.D.K.); sheri.dorsam@ndsu.edu (S.T.D.); tammi.neville@ndsu.edu (T.L.N.); alison.ward@ndsu.edu (A.K.W.); pawel.borowicz@ndsu.edu (P.P.B.); larry.reynolds@ndsu.edu (L.P.R.); joel.caton@ndsu.edu (J.S.C.); 2 Department of Animal Sciences, University of Nebraska-Lincoln, Lincoln, NE 68588, USA; kacie.mccarthy@unl.edu; 3 Central Grasslands Research and Extension Center, North Dakota State University, Streeter, ND 58483, USA; kevin.sedivec@ndsu.edu; 4 Purina Animal Nutrition LLC, Gray Summit, MO 63039, USA; jforcherio@landolakes.com (J.C.F.); rrscott@landolakes.com (R.S.)

**Keywords:** beef heifer, energy supplementation, fetal programming, maternal nutrition, vitamin and mineral supplementation

## Abstract

**Simple Summary:**

Cow-calf operations rely mostly in forage-based systems. Different supplementation strategies are used by beef producers in order to overcome nutrient deficiencies and achieve targeted growth or reproductive performances. This study provides information on the impacts of feeding pregnant replacement heifers with vitamin/mineral and protein/energy supplements on heifer performance and fetal outcomes. Our study shows that moderate rates of gain (achieved via protein/energy supplementation) resulted in fetuses with heavier femurs and reduced liver mass relative to fetal body weight. Moreover, vitamin and mineral supplementation increased fetal liver mass, and vitamin and mineral supplementation combined with moderate gain treatments resulted in greater fetal intestinal weights. These findings indicate that replacement heifer nutrition during early gestation can alter the development of organs in the fetus that are relevant for future offspring performance. Liver and intestines are key organs related to energy metabolism; therefore, this study shows that compensatory mechanisms are in place in the developing conceptus that can alter the growth rate of key metabolic organs possibly in an attempt to increase or decrease energy utilization.

**Abstract:**

Thirty-five crossbred Angus heifers (initial BW = 359.5 ± 7.1 kg) were randomly assigned to a 2 × 2 factorial design to evaluate effects of vitamin and mineral supplementation [VMSUP; supplemented (VTM) vs. unsupplemented (NoVTM)] and different rates of gain [GAIN; low gain (LG), 0.28 kg/d, vs. moderate gain (MG), 0.79 kg/d] during the first 83 d of gestation on dam hormone and metabolic status, fetal tissue and organ mass, and concentration of glucose and fructose in fetal fluids. The VMSUP was initiated 71 to 148 d before artificial insemination (AI), allowing time for mineral status of heifers to be altered in advance of breeding. At AI heifers were assigned their GAIN treatment. Heifers received treatments until the time of ovariohysterectomy (d 83 ± 0.27 after AI). Throughout the experiment, serum samples were collected and analyzed for non-esterified fatty acids (NEFA), progesterone (P4), insulin, and insulin-like growth factor 1 (IGF-1). At ovariohysterectomy, gravid reproductive tracts were collected, measurements were taken, samples of allantoic (ALF) and amniotic (AMF) fluids were collected, and fetuses were dissected. By design, MG had greater ADG compared to LG (0.85 vs. 0.34 ± 0.04 kg/d, respectively; *p* < 0.01). Concentrations of NEFA were greater for LG than MG (*p* = 0.04) and were affected by a VMSUP × day interaction (*p* < 0.01), with greater concentrations for NoVTM on d 83. Insulin was greater for NoVTM than VTM (*p* = 0.01). A GAIN × day interaction (*p* < 0.01) was observed for IGF-1, with greater concentrations for MG on d 83. At d 83, P4 concentrations were greater for MG than LG (GAIN × day, *p* < 0.01), and MG had greater (*p* < 0.01) corpus luteum weights versus LG. Even though fetal BW was not affected (*p* ≥ 0.27), MG fetuses had heavier (*p* = 0.01) femurs than LG, and VTM fetuses had heavier (*p* = 0.05) livers than those from NoVTM. Additionally, fetal liver as a percentage of BW was greater in fetuses from VTM (P = 0.05; 3.96 ± 0.06% BW) than NoVTM (3.79 ± 0.06% BW), and from LG (*p* = 0.04; 3.96 ± 0.06% BW) than MG (3.78 ± 0.06% BW). A VMSUP × GAIN interaction was observed for fetal small intestinal weight (*p* = 0.03), with VTM-MG being heavier than VTM-LG. Therefore, replacement heifer nutrition during early gestation can alter the development of organs that are relevant for future offspring performance. These data imply that compensatory mechanisms are in place in the developing conceptus that can alter the growth rate of key metabolic organs possibly in an attempt to increase or decrease energy utilization.

## 1. Introduction

Dam nutrition during the early stages of pregnancy may have metabolic and physiological effects on the fetus [1,2,3], with the potential to alter the developmental trajectory of offspring later in life [4,5,6,7,8]. Recent studies demonstrate that maternal nutrient restriction (60% of NRC requirements) during the first 50 d of pregnancy affect the transcript abundance of genes associated with tissue metabolism, accretion, and function in fetal liver, muscle, and cerebrum [2]. Maternal plane of nutrition and day of gestation have altered concentrations of key metabolic fuels in allantoic and amniotic fluids, mRNA expression of utero-placental glucose and cationic AA transporters, and the location and abundance of these transporters in beef heifer utero-placental tissues [9,10,11].

To continue investigating the effects of maternal nutrition in fetal development outcomes, we developed a research model where beef heifers were subjected to a vitamin and mineral supplementation and two rates of gain achieved via protein/energy supplements (both within the range of gain observed in commercial production systems) during the first 83 d of gestation. We speculate that strategic supplementation may modulate rates of gain and by doing so potentially affect offspring growth in utero and postnatally. Currently, there is a large variation in vitamin/mineral [12] and protein/energy [13,14] supplementation strategies in beef operations; thus, the beef industry would benefit from the understanding of the effects that vitamin and mineral supplementation and different rates of gain have on maternal hormone and metabolite responses during the first trimester of gestation.

Previously [15], we reported that concentrations of His, Asp, and various neutral AA were greater in the allantoic fluid from heifers fed a vitamin and mineral supplement, while concentrations of Arg, Cys, and Asp were increased in the allantoic fluid of heifers gaining 0.79 kg/d (moderate gain) compared with those on gaining 0.28 kg/d (low gain). Along with glucose and fructose, amino acids play a critical role for establishment and maintenance of pregnancy [3]. Glucose is the primary energy substrate for the placenta and fetus and is essential for normal fetal metabolism and growth [16]. Fructose is the main carbohydrate in plasma and fetal fluids of mammals and may induce cell proliferation via activation of the mammalian target of rapamycin pathway [3,17]. Thus, one of the goals of this study is to investigate how glucose and fructose concentrations in fetal fluids are affected by nutritional management strategies.

Diniz et al. [18] observed that maternal vitamin and mineral supplementation and rate of gain affected placental expression of energy metabolism and transport-related genes using the same subset of animals from our study. Findings of Diniz et al. [18] and Menezes et al. [15] suggest that fetal growth may be impacted using our research model. Thus, a third goal of this study was to evaluate fetal growth and organ mass. Therefore, we hypothesized that maternal vitamin and mineral supplementation and moderate rates of gain would positively affect heifer performance, hormonal and metabolic status, and result in increased concentrations of glucose and fructose in allantoic and amniotic fluids and increase fetal growth and organ mass.

## 2. Materials and Methods

### 2.1. Ethics Statement

All animal procedures were approved by the North Dakota State University Institutional Animal Care and Use Committee (#A19012).

### 2.2. Animals, Experimental Design, Housing and Diet

Crossbred Angus heifers (*n* = 72; 11 to 12 months of age, initial BW = 359.5 ± 7.1 kg) were initially housed at the Central Grasslands Research Extension Center (Streeter, ND), where they were randomly assigned to one of 2 vitamin and mineral supplementation treatments [VMSUP; supplemented (VTM, *n* = 36) vs. unsupplemented (NoVTM, *n* = 36)], thus allowing time for mineral status of heifers to be altered in advance of breeding. Therefore, NoVTM heifers were submitted to a mineral depletion period of at least 71 days, as explained further in the text. Heifers were group-fed and diets were delivered once daily via total mixed ration (TMR) and consisted of triticale hay, corn silage, modified distillers grains plus solubles, ground corn, and if indicated by treatment, vitamin and mineral premix (delivered at a 0.45 kg•heifer^−1^•day^−1^ to target 113 g of product). The vitamin and mineral premix provided vitamins A, D, and E and macro and trace minerals to meet 110% of the requirements specified by the NASEM [19] and consisted of ground corn and a loose mineral supplement (Purina Wind & Rain Storm All-Season 7.5 Complete, Land O’Lakes, Inc., Arden Hills, MN; Table 1). Heifers in the NoVTM treatment received the ground corn carrier at 0.45 kg•heifer^−1^•day^−1^.

**Figure 1:**
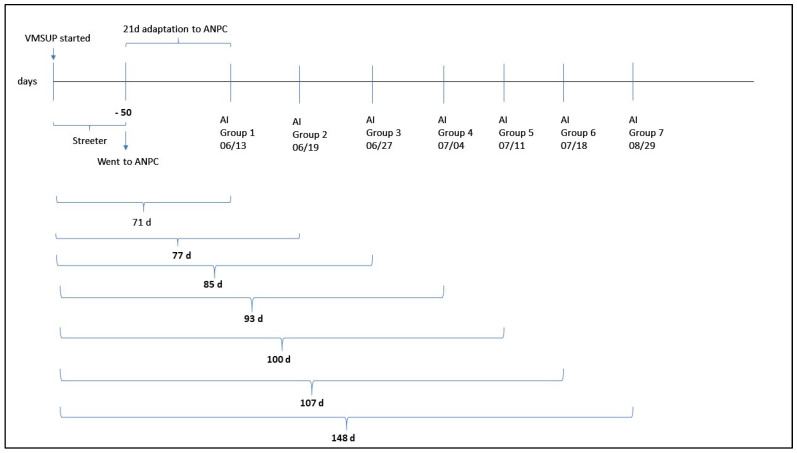
Feeding timeline for vitamin and mineral supplementation [VMSUP; supplemented (VTM) vs. unsupplemented (NoVTM)]. AI: Artificial Insemination; ANPC: Animal Nutrition and Physiology Center (Fargo, ND, USA).

Fifty days after initiation of the VMSUP factor, heifers were transported approximately 227 km and housed at the North Dakota State University Animal Nutrition and Physiology Center (ANPC; Fargo, ND, USA). At ANPC, heifers were randomly allocated in 12 group-pens (23.7 m^2^) with 6 heifers per pen. Therefore, a same pen housed VTM and NoVTM heifers. Heifers were individually fed daily in an electronic head gate facility (American Calan; Northwood, NH, USA), and continued to receive their respective VMSUP treatments until the time of artificial insemination (AI). At this facility, the VTM heifers received a pelleted vitamin and mineral supplement fed at a 0.45 kg•heifer^−1^•day^−1^, consisting of 113 g of the vitamin and mineral supplement and formulated to deliver similar levels of vitamins and minerals that were fed pre-breeding, while the NoVTM heifers received a pelleted carrier product fed at a 0.45 kg•heifer^−1^•day^−1^ with no added vitamins or minerals. The duration of time VTM and NoVTM heifers received their treatments varied according to the AI group they were assigned (i.e., treatments were initiated on the same calendar day, but AI occurred over seven AI group timepoints due to logistical constraints; Figure 1). Therefore, the VMSUP factor was initiated 71 to 148 d before AI. The rationale for the mineral feeding timeline is threefold: (1) because of various mineral stores in the body, mineral status as a whole takes time to alter; therefore we targeted at least 70 d on the VMSUP factor to allow time for mineral status to be altered in advance of breeding so effects would be present on offspring from conception through the gestational target; (2) this is a production-relevant scenario as producers typically either feed a supplement or not, and would likely not decide to stop or start feeding a VTM supplement at the time of breeding; (3) to successfully manage workflow for the experiment we needed to control the number of heifers bred and tissue collections conducted on a single day. Based on this rationale, the duration of pre-breeding time on the VTM treatment varied according to their breeding group with a range of 71 to 148 days before AI. The breeding group was taken into consideration in our statistical analysis and had no influence in any of the response variables. All heifers were subjected to a 7-d CO-Synch + CIDR estrus synchronization protocol [20], and AI bred to female sexed semen from a single sire.

At AI (d 0 of our experiment), heifers were assigned randomly to either LG or MG treatments—within their respective VMSUP factor—completing the factorial arrangement of treatments. Heifers that received a protein/energy supplementation (a proprietary blend of ground corn, dried distillers grains plus solubles, wheat midds, fish oil, urea, and ethoxyquin; fed at the rate of 0.58% of BW as-fed daily) were targeted to gain 0.79 kg/d (MG), while heifers that did not receive the protein/energy supplementation were targeted to gain 0.28 kg/d (LG). All heifers received a basal diet made up by prairie grass hay, corn silage, and dried distillers grains plus solubles (Table 2). If indicated by treatment, supplements were top dressed over the basal diet.

Pregnancy diagnosis was performed 35 days after AI, and fetal sex was determined on d 65 after AI using transrectal ultrasonography [21]. After pregnancy diagnosis and fetal sexing, 35 of the 72 heifers originally enrolled were gestating female fetuses and remained in the experiment, in the following treatment combinations: (1) No vitamin and mineral supplement, low gain (NoVTM-LG; *n* = 9); (2) No vitamin and mineral supplement, moderate gain (NoVTM-MG; *n* = 9); (3) Vitamin and mineral supplement, low gain (VTM-LG; *n* = 9); (4) Vitamin and mineral supplement, moderate gain (VTM-MG; *n* = 8). Heifers were weighed at weekly intervals and individual feed intake adjusted during the course of the study to achieve targeted BW gains. The targeted daily gains proposed in our research model were both within the range observed in normal production scenarios, reflecting what has been observed in unsupplemented and supplemented grazing beef cattle [22,23,24]. Another important consideration regarding forage-based production systems is that seasonal variations in forage quantity and quality affect nutrient availability in such a manner that forage diets may not always meet nutritional requirements, including mineral requirements (No-VTM-LG diet, Table 3). In this context, the supplementation strategies used in this study (VTM and MG) were designed to overcome potential deficiencies and achieve targeted production goals for growth and reproductive performance of commercial beef herds. Our treatments were applied until the experiment endpoint of d 83 ± 0.27 after breeding, when heifers were ovariohysterectomized.

### 2.3. Feed Analysis

Diet TMR samples were collected weekly throughout the experiment and composited over the feeding period. The composited sample was dried in a 55 °C oven and ground to pass through a 2 mm screen. Samples were analyzed for dry matter, ash, N (Kjehldahl method), Ca, P, and ether extract by standard procedures (AOAC, 1990). Crude protein was determined by multiplying N by 6.25. Neutral detergent fiber and acid detergent fiber concentrations were determined by the modified method of Van Soest et al. [25] using a fiber analyzer (Ankom Technology Corp., Fairport, NY, USA). The TMR was also analyzed for concentrations of Cu, Zn, Mo, Fe, and S using inductively coupled plasma optical emission spectroscopy and concentrations of Co and Se via inductively coupled plasma mass spectrometry by the Veterinary Diagnostic Laboratory at Michigan State University.

### 2.4. Blood Sample Collections and Analysis

Serum samples from the jugular vein were collected into 10 mL serum vacutainer tubes (Becton Dickinson Co., Franklin Lakes, NJ, USA) at the time of VMSUP factor initiation (i.e., 71 to 148 d before AI), controlled internal drug release (CIDR) insertion (9 days before AI), and on d 14, 28, 42, 56, 70, and 83 ± 0.27 after AI to determine concentrations of glucose, non-esterified fatty acids (NEFA), and P4. Serum samples collected at VMSUP factor initiation, at the time of CIDR insertion, and at the time of ovariohysterectomy (d 83 ± 0.27 after breeding) were further analyzed to determine concentrations of insulin and insulin-like growth factor 1 (IGF-1). All blood samples were collected prior to the morning feeding, placed on ice, centrifuged at 1500× *g* at 4 °C for 20 min, and stored in plastic vials at −20 °C until further analysis.

Samples were analyzed for glucose and NEFA using the Synergy H1 Microplate Reader (Biotek, Winooski, VT, USA) with the Infinity Glucose Hexokinase Kit (Thermo Scientific, Waltham, MA, USA) and NEFA-C Kit (WAKO Chemicals, Inc., Richmond, VA, USA). The intra- and inter-assay CV were 2.87 and 6.80%, respectively, for glucose and 2.96 and 17.46%, respectively, for NEFA.

Concentrations of P4, insulin [26,27], and IGF-1 [28,29,30] were analyzed by competitive chemiluminescent immunoassay using the Immulite 1000 (Siemens, Los Angeles, CA, USA). A 50 µL sample of maternal serum and all controls were analyzed in duplicate. Lyphochek Immunoassay Plus Control Levels 1, 2 and 3 were purchased from Biorad (cat. No 370X) to provide quality controls for P4 assays. For P4, low, intermediate and high control values were measured within expected ranges (0.94 ± 0.12, 8.44 ± 0.94, and 19.6 ± 0.54 ng/mL, respectively). The intra-assay CV was 4.15%, and the inter-assay CVs for the controls were 12.9, 11.2, and 2.8%, respectively. For insulin, low and high controls from Siemens were utilized to validate the assay (8.03 ± 0.47 and 49.6 ± 1.41 uIU/mL, respectively), and intra- and inter-assay CVs were 5.13 and 4.33%, respectively. For IGF-1, low and high controls from Siemens were measured within expected ranges (65.2 ± 2.26 and 204 ± 8.49 ng/mL, respectively), and the intra-assay CV was 2.59%, and the inter-assay CV was 3.81%.

### 2.5. Utero-Placental and Fetal Tissues Collection and Analysis

Ovariohysterectomy procedures were conducted at d 83 ± 0.27 of gestation to collect utero-placental tissues as previously described by McLean et al. [31]. Immediately following collection, weights of the gravid uterus, ovaries, and CL were recorded. The fetus was removed, weighed, and the side of the fetus was photographed using Omni ASH Digital Videoscope (ASH, Kildare, Ireland) system calibrated to take images and measurements in microns at any magnification. The calibrated ASH images were analyzed using image analysis software (Image Pro Premier v. 9.3, Media Cybernetics, Rockville, MD, USA): crown-rump length as the straight line from the top of the head (crown) to the head of the tail, curved crown-rump length as the line along the body curve from the top of the head to the head of the tail, eye diameter, nose to poll (crown), body depth at front shoulder (wither ends), and body depth at the umbilicus (umbilicus to the lower back at the height of L vertebra).

Subsequently, the fetus was dissected, and the fetal liver, heart, intestine, pancreas, hindlimb, brain, and ovaries were collected. Fetal organs were individually weighed, processed, and stored at −80 °C for further analysis.

Allantoic (ALF) and amniotic (AMF) fluids were collected using a 22-gauge needle (Medtronic, Minneapolis, MN, USA) to penetrate the respective fetal membranes, and fluids were suctioned with a 10 mL syringe [2,15]. Aliquots of the fluids were placed in 2 mL microtubes, snap frozen on dry ice, and stored at −80 °C for subsequent glucose and fructose analyses. Samples were analyzed for glucose using the Synergy H1 Microplate Reader (Biotek, Winooski, VT, USA) with the Infinity Glucose Hexokinase Kit (Thermo Scientific, Waltham, MA, USA). To determine fructose concentration, frozen samples of ALF and AMF were thawed at room temperature, centrifuged for 5 min at 14,000× *g* and 18 °C to remove any debris that would interfere with the assay. The thawed, pre-cleared ALF supernatants were diluted 1:80 and AMF samples diluted 1:10 with 18 mΩ water. The EnzyChrom Fructose Assay Kit (EFRU-100; BioAssay Systems, Hayward, CA, USA) was utilized on a Synergy H1 Microplate Reader (BioTek) at 565 nm and 25 °C. Final results were reported in µmol of fructose. Pooled samples of ALF and AMF were used as controls (intra-assay CV = 3.40 and 4.44% for ALF and AMF, respectively; inter-assay CV = 16.06% for ALF and 9.31% for AMF).

### 2.6. Statistical Analysis

Data were analyzed using the MIXED procedure of SAS 9.4 (SAS Inst. Inc., Cary, NC, USA). Body weight, blood hormones and metabolites were analyzed as repeated measures with VMSUP, GAIN, day, and their interactions as fixed effects and heifer as a random effect. The covariate structures were tested, and the structure with the lowest Akaike information criterion/Bayesian information criterion was used for all variables analyzed. Maternal performance, uterine and fetal measurements, and glucose and fructose concentrations in ALF and AMF were analyzed using the MIXED procedures of SAS with VMSUP, GAIN, and a VMSUP × GAIN interaction as fixed effects and heifer as a random effect. Means were separated using the PDIFF procedure of SAS with a Tukey–Kramer adjustment. Results are presented as least squares means (LSMEANS) with their standard errors. For all analyses, heifer was considered the experimental unit and *p*-values ≤ 0.05 were considered significant.

## 3. Results

### 3.1. Performance and Intake

**Figure 2:**
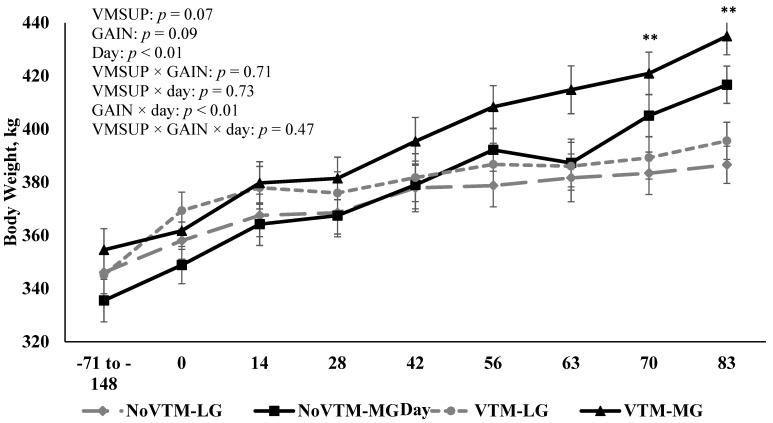
Effect of vitamin and mineral supplementation [VMSUP; supplemented (VTM) vs. unsupplemented (NoVTM); from pre-breeding to d 83 of gestation] and two different rates of gain [GAIN; low gain (LG), 0.28 kg/d, vs. moderate gain (MG), 0.79 kg/d; from breeding to d 83 of gestation] on beef heifer body weight. Because of the seven breeding groups the heifers were assigned to, the extent on which VTM heifers received their treatments varied according to their breeding date. Therefore, the VMSUP factor started within a range of 71 to 148 days before artificial insemination (AI). The AI was considered the d 0 of the experiment—where heifers were assigned to their GAIN treatments—completing our factorial design. NoVTM-LG (*n* = 9): No vitamin and mineral supplement, low gain; NoVTM-MG (*n* = 9): No vitamin and mineral supplement, moderate gain; VTM-LG (*n* = 9): Vitamin and mineral supplement, low gain; VTM-MG (*n* = 8): Vitamin and mineral supplement, moderate gain. A GAIN × day interaction (*p* < 0.01) was observed for body weight, which was similar between LG and MG from project initiation until d 70; and was greater (*p* ≤ 0.04) for MG compared with LG on d 70 and 83, as indicated by **. Values are least squares means, with error bars depicting standard error.

Average daily gain (ADG) was not affected by VMSUP (*p* = 0.72) or by a VMSUP × GAIN interaction (*p* = 0.35; Table 4). As designed, ADG was greater (*p* < 0.01) for MG heifers (0.85 ± 0.04 kg/d) than LG heifers (0.34 ± 0.04 kg/d; Table 4). Consequently, MG heifers were heavier than LG heifers towards the end of the trial, as evidenced by a GAIN × day interaction (*p* < 0.01; Figure 2), with a final BW of 426.8 ± 6.1 vs. 387.6 ± 5.7 kg for MG vs. LG heifers, respectively.

To achieve targeted gains, total dry matter intake (DMI) was greater (*p* < 0.01) for MG (7.72 ± 0.21 kg/d) than LG (4.98 ± 0.19 kg/d) heifers (Table 4). Feed efficiency (gain:feed) was affected by GAIN, with greater values (*p* < 0.01) for MG than LG heifers (Table 3). Total DMI, DMI of TMR, and DMI of the protein and energy supplement were not influenced by VMSUP (*p* ≥ 0.49) or the VMSUP × GAIN interaction (*p* ≥ 0.08).

### 3.2. Blood Hormones and Metabolites

There were no differences in plasma glucose concentrations due to main effects or interactions (*p* ≥ 0.08). Concentrations of glucose averaged 3.96 ± 0.15, 4.18 ± 0.14, 3.86 ± 0.19, and 4.25 ± 0.17 mmol/L for NoVTM-LG, NoVTM-MG, VTM-LG, and VTM-MG, respectively.

**Figure 3:**
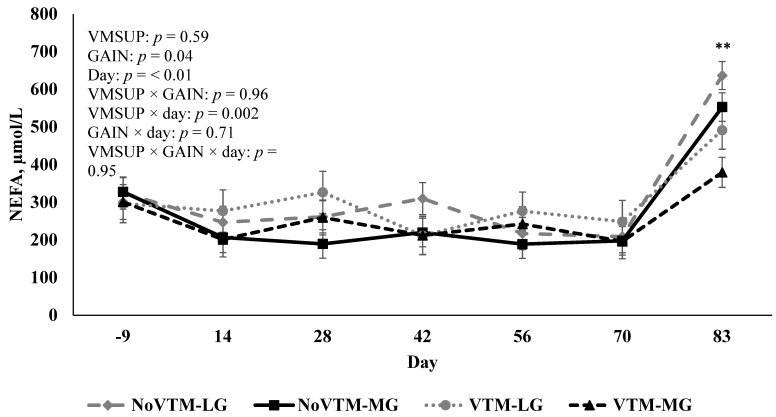
Effect of vitamin and mineral supplementation [VMSUP; supplemented (VTM) vs. unsupplemented (NoVTM); from pre-breeding to d 83 of gestation] and two different rates of gain [GAIN; low gain (LG), 0.28 kg/d, vs. moderate gain (MG), 0.79 kg/d; from breeding to d 83 of gestation] on blood non-esterified fatty acids (NEFA) concentrations. NoVTM-LG (*n* = 9): No vitamin and mineral supplement, low gain; NoVTM-MG (*n* = 9): No vitamin and mineral supplement, moderate gain; VTM-LG (*n* = 9): Vitamin and mineral supplement, low gain; VTM-MG (*n* = 8): Vitamin and mineral supplement, moderate gain. A VMSUP × Day interaction (*p* = 0.002) was observed for NEFA, being similar for NoVTM and VTM from d −9 to 70; and greater (*p* = 0.02) for VTM than NoVTM on day 83 as indicated by **. Values are least squares means, with error bars depicting standard error.

Concentrations of NEFA were affected by GAIN (*p* = 0.04), with lower concentrations for MG (262.54 ± 15.28 µmol/L) than LG (309.57 ± 17.03 µmol/L) heifers throughout the experiment. Further, there was a VMSUP × day interaction (*p* = 0.002) for concentrations of NEFA, with greater concentrations for NoVTM than VTM heifers (*p* = 0.02) at d 83 of gestation (Figure 3).

**Figure 4:**
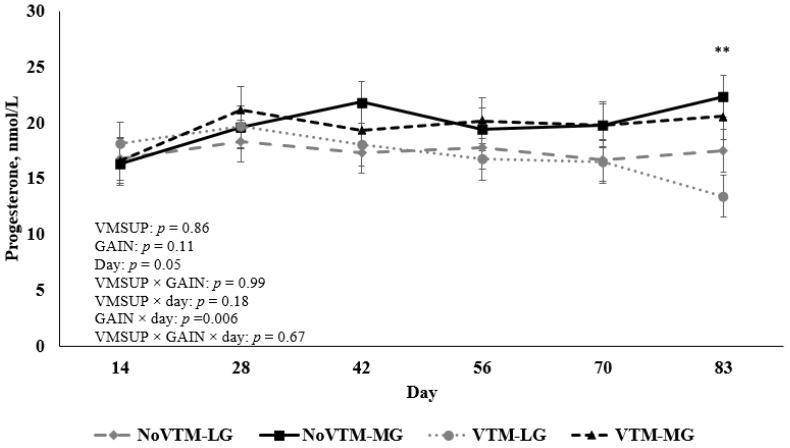
Effect of vitamin and mineral supplementation [VMSUP; supplemented (VTM) vs. unsupplemented (NoVTM); from pre-breeding to d 83 of gestation] and two different rates of gain [GAIN; low gain (LG), 0.28 kg/d, vs. moderate gain (MG), 0.79 kg/d; from breeding to d 83 of gestation] on progesterone (P4) concentrations. NoVTM-LG (*n* = 9): No vitamin and mineral supplement, low gain; NoVTM-MG (*n* = 9): No vitamin and mineral supplement, moderate gain; VTM-LG (*n* = 9): Vitamin and mineral supplement, low gain; VTM-MG (*n* = 8): Vitamin and mineral supplement, moderate gain. A rate of gain × Day interaction (*p* = 0.006) was observed for P4 concentrations, which were similar between LG (low gain, 0.28 kg/d) and MG (moderate gain, 0.79 kg/d) on d 14 to 70; and were greater (*p* = 0.05) for MG compared with LG on d 83, as indicated by **. Values are least squares means, with error bars depicting standard error.

A GAIN × day interaction (*p* < 0.01; Figure 4) was observed for concentrations of P4, with greater concentrations (*p* = 0.05) for MG than LG heifers on d 83 of gestation (21.44 ± 1.36 vs. 15.44 ± 1.31 nmol/L, respectively). Concentrations of P4 were not affected by the main effects of VMSUP (*p* = 0.86) or GAIN (*p* = 0.11) nor other interactions (*p* ≥ 0.18).

Concentrations of insulin in maternal serum were not affected by any of the (*p* ≥ 0.09; Table 5) interactions tested nor by the main effect of GAIN (*p* = 0.16). Concentrations of insulin increased throughout the experiment (*p* < 0.01), with greater concentrations for all groups of heifers at d 83 of gestation (51.07 ± 2.76 pmol/L) compared to samples collected before VMSUP initiation (26.11 ± 2.76 pmol/L) and at the time of CIDR insertion (34.29 ± 2.76 pmol/L). Further, a VMSUP effect was observed (*p* = 0.01), with greater concentrations for NoVTM (43.16 ± 2.97 pmol/L) than VTM (31.15 ± 3.12 pmol/L) heifers.

A GAIN × day interaction (*p* < 0.01; Table 5) was observed for concentrations of IGF-1, with greater concentrations for MG (18.61 ± 0.85 nmol/L) than LG (17.26 ± 0.82 nmol/L) heifers at d 83 of gestation. Concentrations of IGF-1 were not affected by the main effects of VMSUP (*p* = 0.49) or GAIN (*p* = 0.25) nor any of the other interactions tested (*p* ≥ 0.30).

### 3.3. Maternal Reproductive Tract, Fetal Characteristics, and Glucose and Fructose Concentrations in ALF and AMF on d 83 of Gestation

Gravid and empty uterine weight and mean diameter of the 3 largest placentomes were not affected by VMSUP (*p* ≥ 0.35), GAIN (*p* ≥ 0.43), or their interaction (*p* ≥ 0.16; Table 6). However, CL weight and diameter were influenced by GAIN (*p* < 0.01 and *p* = 0.02, respectively), both being greater for MG than LG heifers.

Fetal BW was not affected by main effects of VMSUP or GAIN (*p* ≥ 0.27) or their interaction (*p* = 0.28; Table 7). However, fetal intestinal weight was affected by a VMSUP × GAIN interaction (*p* = 0.03), where intestine from VTM-MG fetuses were heavier than those from VTM-LG (*p* = 0.04), with all other treatments being similar (*p* ≥ 0.08). This effect was not observed (*p* ≥ 0.15) when fetal intestinal mass was evaluated as a percentage of fetal BW. No other fetal organ weights (either absolute mass or as a percentage of fetal BW) were affected by a VMSUP × GAIN interaction (*p* ≥ 0.15). However, femur weight was affected by GAIN, where fetuses from MG had heavier (*p* = 0.01) femurs than those from LG. Fetal liver weight was heavier (*p* = 0.05) for fetuses from VTM than NoVTM when analyzed as absolute weight, and when analyzed as a percentage of fetal BW. Interestingly, fetal liver weight as a percentage of fetal BW was greater (*p* = 0.04) for LG than MG heifers. Fetal heart, pancreas, hindlimb, brain, and ovaries (either absolute mass or as a percentage of fetal BW) were not affected by main effects of VMSUP (*p* ≥ 0.14), GAIN (*p* ≥ 0.07), or their interaction (*p* ≥ 0.08). Fetal body measurements (crown rump length, curved crown rump length, eye diameter, nose to poll, body depth at front shoulder, and body depth at umbilicus; Table 6) were not affected by VMSUP (*p* ≥ 0.19), GAIN (*p* ≥ 0.20), or their interaction (*p* ≥ 0.06).

Concentrations of glucose and fructose in ALF and AMF were not affected (*p* ≥ 0.16 and *p* ≥ 0.27 for glucose and fructose, respectively) by VMSUP, GAIN, or their interaction. Concentrations of glucose averaged 2.61 ± 0.11 and 2.13 ± 0.04 mmol/L in ALF and AMF, respectively; while concentrations of fructose averaged 28.61 ± 1.8 and 4.15 ± 0.14 mmol/L in ALF and AMF, respectively.

## 4. Discussion

Our model achieved the proposed divergence in targeted daily gains. For example, gestating heifers fed the protein and energy supplement (MG) were targeted to gain 0.79 kg/d and had an actual ADG of 0.85 kg/d, whereas unsupplemented heifers (LG) were targeted to gain 0.28 kg/d and had an actual ADG of 0.34 kg/d. We reported that gestating heifers grown at a moderate rate of gain had increased concentrations of IGF-1 and P4, lower concentrations of NEFA throughout the experiment, greater CL weight at d 83 of gestation, and their fetuses had greater fetal femur weight and reduced liver weight (as a percentage of BW) compared with heifers grown at a lower rate of gain. We did not observe differences in concentrations of glucose and fructose in fetal fluids in response to maternal treatments; however, this study demonstrated that providing a vitamin and mineral supplement during the first trimester of gestation resulted in greater fetal liver weight, whereas the interaction between vitamin/mineral and protein/energy supplementation resulted in greater fetal intestinal weight. Findings from this study indicate that response variables that diverged in the dam were also experienced in some way by the fetus. Our results may help to understand how maternal nutritional strategies in early pregnancy can alter the development of organs that are relevant for future offspring performance.

It is well known that levels of metabolic hormones and metabolic factors are regulated by nutritional status. For example, key responses such as follicular development, oocyte quality, maternal-embryo recognition, embryo and fetal development, and pregnancy maintenance have been shown to be affected by nutrition, body condition, and metabolic status of beef heifers and cows [32,33]. In the current experiment, moderate rates of gain during the first 83 d of gestation resulted in larger and heavier CL and greater concentrations of P4 compared to low rates of gain. In a series of four studies involving ultrasonography and ovarian dissection in beef cows, Rocha et al. [34] reported that CL weight and CL area are the variables with the greatest correlation with plasma P4 concentrations. Progesterone is an essential marker of CL activity and plays a critical role on the establishment and maintenance of pregnancy [35]. Further, P4 has a well-established positive role in uterine function by regulating endometrial secretions essential for stimulating and mediating conceptus growth [36] and differentiation throughout early pregnancy in ruminants [34,37]. Therefore, our findings suggest that the increase in maternal P4 concentrations in response to moderate maternal rates of gain may have improved the uterine environment by potentially increasing the flow of nutrients, metabolites, and hormones to the fetus.

Additionally, nutritional plane and rates of gain are associated with IGF-1 levels. Similar to our study, Rodríguez-Sánchez et al. [38] reported that beef heifers fed diets formulated for high rates of gain (0.8 kg/d) had greater concentrations of plasma IGF-1 than heifers fed to achieve moderate rates of gain (0.6 kg/d). Noya et al. [33] observed greater concentrations of IGF-1 in beef cows fed 100% of energy requirements during the first 82 d of gestation compared with cows fed 85% of energy requirements. These authors further demonstrated a greater negative correlation between concentrations of NEFA and IGF-1 [33]. Our data show a similar relationship, with MG heifers having greater concentrations of IGF-1 and reduced NEFA concentrations compared to LG heifers. An important consideration is that IGF-1 plays a critical role in fetal growth [39], which was observed in the current experiment as greater femur weight for fetuses from MG heifers. This finding of an effect on bone growth suggests a potential for enhanced growth of future offspring from heifers managed at moderate rates of gain compared with low rates of gain. Investigation beyond the current evaluation period is warranted to determine whether effects of maternal rate of gain during the first trimester of gestation has subsequent impacts on offspring that extend through gestation to birth and later post-natal periods.

The fact that fetal liver mass (as a % of BW) was increased in fetuses from LG heifers indicates that the liver grew at a greater rate than that of the overall body during the first 83 d of gestation. Studies investigating fetal hepatic and small intestinal in vitro oxygen consumption in pregnant ewes, cows, and their fetuses [40,41,42] have demonstrated that both dams and fetal offspring during gestation can modulate maintenance energy requirements in response to nutrient restriction and realimentation. Even though we did not work with a nutrient restriction model, our treatments were designed to model growth trajectories of grazing heifers receiving or not receiving protein and energy supplement during early gestation. Whole-animal or specific tissue oxygen consumption reflects energy use and mitochondrial function [1]. Thus, our results may be an indication that compensatory mechanisms are in place in the developing conceptus that can alter the growth rate of key metabolic organs possibly in an attempt to enhance utilization of available nutrients. Research is warranted to determine whether alterations in fetal organ weight extend postnatally, and whether postnatal effects would result in altered postnatal metabolic phenotype.

An interesting observation was that heifers receiving VTM had reduced concentrations of NEFA on d 83 compared with heifers not receiving VTM. Leading up to d 83, heifers were handled 2 additional days for surgical preparation and were fasted for 24 h, and water was deprived for 12 h before the d 83 sample collection; therefore, the dramatic increase in NEFA for all heifers on this day was expected. Such stressors may increase the levels of catecholamines and glucocorticoids that stimulate lipolysis [43]. Perhaps our observation indicated that the vitamin and mineral supplementation allowed heifers to better cope with the stresses of handling and fasting. Further, greater NEFA concentration at d 83 of gestation for NoVTM heifers can be interpreted as an indicator of decreased insulin sensitivity, which is characterized by increased concentrations of circulating insulin [44]. In fact, during this experiment, NoVTM heifers had greater concentrations of insulin than VTM heifers. It is important to consider that physical and psychological stressors can result in oxidative stress [45], that compromises cellular integrity, cellular enzymatic activities, immune response, hormone production, and ultimately can lead to insulin resistance [46]. Trace minerals, particularly Se, Zn, Mn, Cu, and Cr, along with vitamin E, are key components of the cellular antioxidant defense. These minerals are cofactors of enzymatic reactions that consume reactive oxygen species and/or are present in the structure of enzymes such as glutathione peroxidase and enzymes of the superoxide dismutase family that play a significant role as scavengers of free radicals, whereas vitamin E is the first line of defense against peroxidation of vital phospholipids [47]. Thus, it is possible that the lower concentrations of NEFA and insulin observed in VTM heifers at d 83 might be a function of these micronutrients attenuating a stress response that was manifested by 3 consecutive days of heifers being handled in preparation of surgery on d 83. Future experiments, therefore, should evaluate biomarkers of oxidative stress and other indicators of stress response to our VTM treatments.

Other novel finding of the present study was the greater fetal liver weight (absolute weight and as a percentage of BW) of fetuses from VTM heifers, indicating that a vitamin and mineral supplementation at prebreeding and early gestation play a role in fetal liver development. Further, we observed that heifers supplemented with vitamins/minerals and protein/energy presented greater fetal intestinal mass than those receiving a VTM supplement but not supplemented with protein/energy, implying a greater intestinal functional area. However, small intestine growth and development is affected not only during organogenesis, but also during the perinatal and neonatal periods [48]. Therefore, post-natal evaluation of intestinal morphometry and capacity of nutrient absorption are warranted.

In a previous study [15] we reported increased concentrations of amino acids in maternal serum and ALF of beef heifers at d 83 of gestation in response to VTM supplementation and rate of gain of 0.79 kg/d. Amino acids, along with glucose and fructose are key metabolic fuels to the fetus [3]; therefore, one of the hypotheses of this study was that a maternal vitamin and mineral supplementation and moderate rates of gain would result in increased concentrations of glucose and fructose in ALF and AMF. Contrary to what we expected, no differences were observed in the concentration of the afore mentioned metabolites. A recent study [2] showed that glucose concentrations tended to be greater in ALF of heifers fed a control diet (100% of NRC requirements to gain 0.45 kg/d) when compared to heifers fed a restricted diet (60% of NRC requirements). Further, data from the same study [2] demonstrate that glucose and fructose concentrations were greater in AMF of control than restricted heifers. Therefore, we can speculate that the lack of difference in glucose and fructose concentrations in both ALF and AMF may be due to an adequate maternal nutrient supply, since the current experiment did not have a nutrient restricted diet; and it may further be explained by the fact that glucose and fructose concentrations are interrelated, since production of the later depends on supply of the former.

## 5. Conclusions

In summary, vitamin/mineral and protein/energy supplementation of pregnant beef heifers are advantageous as it overcomes potential mineral deficiencies in forage-based diets and are a solid strategy to achieve targeted production goals for growth and reproductive performance of commercial beef herds. In addition, results of this study demonstrate that (1) moderate rates of gain result in heavier fetal femur weight and reduced fetal liver proportional mass; (2) vitamin and mineral supplementation results in greater fetal liver absolute and proportional mass; and (3) vitamin and mineral supplementation combined with moderate rates of gain resulted in greater fetal intestinal mass than VTM supplementation combined with low rates of gain. These preliminary data are interpreted to imply that fetuses have the ability to alter the growth rate of key metabolic organs in an attempt to increase or decrease energy utilization. Further research is necessary to study energy expenditure of fetal tissues, which could result in the development of nutritional strategies to improve mitochondrial function and the efficiency of energy utilization in the future offspring.

## Figures and Tables

**Table 1:** Micronutrient composition of vitamin and mineral (VTM) supplement ^1^ provided to beef heifers during the first trimester of gestation; company guaranteed analysis.

Item	Assurance Levels	
Minerals ^1^	Min	Max
Calcium, g/kg of DM	135.0	162.0
Phosphorus, g/kg of DM	75.0	-
Sodium Chloride, g/kg of DM	180.0	216.0
Magnesium, g/kg of DM	10.0	-
Potassium, g/kg of DM	10.0	-
Manganese, mg/kg of DM	3600.0	-
Cobalt, mg/kg of DM	12.0	-
Copper, mg/kg of DM	1200.0	-
Iodine, mg/kg of DM	60.0	-
Selenium, mg/kg of DM	27.0	-
Zinc, mg/kg of DM	3600.0	-
Vitamins ^2^, IU/kg of DM		
A	661,500.0	
D	66,150.0	
E	661.5	

^1^ Purina Wind and Rain Storm All Season 7.5 Complete Mineral (Land O’Lakes, Inc., Arden Hills, MN, USA); Ingredients: Dicalcium Phosphate, Monocalcium Phosphate, Processed Grain By-Products, Plant Protein Products, Calcium Carbonate, Molasses Products, Salt, Mineral Oil, Potassium Chloride, Magnesium Oxide, Ferric Oxide, Vitamin E Supplement, Vitamin A Supplement, Lignin Sulfonate, Cobalt Carbonate, Manganese Sulfate, Ethylenediamine Dihydroiodide, Zinc Sulfate, Copper Chloride, Vitamin D3 Supplement, Natural and Artificial Flavors, Sodium Selenite. Post-breeding, the VTM supplement was delivered as a pelleted product fed at 0.45 kg•heifer^−1^•day^−1^ (consisting of 113 g of a mineral and vitamin supplement, formulated to deliver similar levels of vitamins and minerals that were fed pre-breeding and 337 g of a carrier). ^2^ Ingredients: Vitamin A Supplement (proprietary), Vitamin E Supplement (proprietary), Vitamin D3 Supplement (proprietary).

**Table 2:** Nutrient composition of basal diet and supplements provided to replacement beef heifers during the first trimester of gestation.

Chemical Composition	Basal Diet ^1^	Supplements		
		NoVTM ^2^	VTM ^3^	Protein/Energy ^4^
Dry Matter, %	53.0	86.6	89.6	87.7
Ash, % DM	11.5	5.3	25.1	2.4
Crude Protein, % DM	9.9	15.6	14.8	17.5
Neutral Detergent Fiber, % DM	65.9	41.9	27.6	19.4
Ether Extract, % DM	1.5	0	0	9.1
Non-Fiber Carbohydrates, % DM	11.1	37.2	32.5	51.6
Mineral Content				
Calcium, g/kg DM	5.74	2.47	50.62	0.30
Phosphorus, g/kg DM	2.05	8.94	22.82	4.59
Sodium, g/kg DM	0.26	0.12	19.44	0.24
Magnesium, g/kg DM	2.83	4.47	5.20	1.96
Potassium, g/kg DM	15.81	14.22	13.15	6.05
Sulfur, g/kg DM	2.25	2.41	4.84	2.57
Manganese, mg/kg DM	121.2	103.9	953.4	26.0
Cobalt, mg/kg DM	0.36	0.14	3.38	0.05
Copper, mg/kg DM	4.8	13.7	285.8	3.6
Selenium, mg/kg DM	0.3	0.4	7.0	0.3
Zinc, mg/kg DM	28.4	130.2	1051.8	35.0

^1^ Proportion of ingredients: prairie grass hay (55%), corn silage (38%), and dried distillers grains plus solubles (7%). ^2^ NoVTM: No vitamin mineral supplement was a pelleted product fed at a 0.45 kg•heifer^−1^•day^−1^ with no added vitamin and mineral supplement. ^3^ VTM: Vitamin mineral supplement was a pelleted product fed at a 0.45 kg•heifer^−1^•day^−1^ (consisting of 113 g of a vitamin and mineral supplement, formulated to deliver similar levels of vitamins and minerals that were fed pre-breeding and 337 g of a carrier).^4^ Blend of ground corn, dried distillers grains plus solubles, wheat midds, fish oil, urea, and ethoxyquin fed at rate to achieve targeted gain of 0.79 kg/d for MG treatment.

**Table 3:** Dietary intake of macro and trace minerals and values recommended by the BCNRM (2016; Nutrient Requirements of Beef Cattle).

	Dietary Treatments				BCNRM (2016) Requirements
Composition	NoVTM-LG ^1^	NoVTM-MG ^2^	VTM-LG ^3^	VTM-MG ^4^	
Macrominerals					
Ca, g/d	25.70	32.98	48.04	50.42	26.30
P, g/d	12.32	23.57	19.07	28.61	14.71
Mg, g/d	13.94	21.03	15.72	20.40	9.80
K, g/d	73.67	103.82	81.42	98.08	48.99
Na, g/d	1.17	1.93	9.09	9.64	5.72
S, g/d	10.63	18.22	12.78	18.47	12.25
Microminerals					
Co, mg/d	1.61	2.12	3.10	3.31	1.22
Cu, mg/d	26.02	38.58	138.34	146.82	81.65
Mn, mg/d	562.73	754.58	967.92	1056.12	326.59
Se, mg/d	1.45	2.38	4.27	4.94	0.82
Zn, mg/d	173.11	273.86	560.82	637.53	244.94

^1^ NoVTM-LG: Total mixed ration, no vitamin and mineral supplement, low gain (0.28 kg/d). ^2^ NoVTM-MG: Total mixed ration, no vitamin and mineral supplement, moderate gain (0.79 kg/d). ^3^ VTM-LG: Total mixed ration, vitamin and mineral supplement, low gain (0.28 kg/d). ^4^ VTM-MG: Total mixed ration, vitamin and mineral supplement, moderate gain (0.79 kg/d). The total mixed ration was made by prairie grass hay (55%), corn silage (38%), and dried distillers grains plus solubles (7%). No vitamin mineral supplement (NoVTM) was a pelleted product fed at a 0.45 kg•heifer^−1^•day^−1^ with no added vitamin and mineral supplement. Vitamin mineral supplement (VTM) was a pelleted product fed at a 0.45 kg•heifer^−1^•day^−1^ (consisting of 113 g of a vitamin and mineral supplement, formulated to deliver similar levels of vitamins and minerals that were fed pre-breeding and 337 g of a carrier). A blend of ground corn, dried distillers grains plus solubles, wheat midds, fish oil, urea, and ethoxyquin was fed at rate to achieve targeted gain of 0.79 kg/d for moderate gain treatment (MG).

**Table 4:** Performance and dry matter intake (DMI) of beef heifers during the first trimester of gestation as influenced by vitamin and mineral supplementation [VMSUP; not supplemented (NoVTM) or supplemented (VTM)] and two different rates of gain [GAIN; low rate, 0.28 kg/d (LG) or moderate rate, 0.79 kg/d (MG)].

	NoVTM ^1^		VTM ^2^			*p*-Values		
Item	LG	MG ^3^	LG	MG ^3^	SEM ^4^	VMSUP	GAIN	VMSUP × GAIN
Average Daily Gain ^5^, kg/d	0.35	0.82	0.32	0.88	0.04	0.72	<0.01	0.35
Total Mixed Ration DMI, kg/d	4.24	5.53	4.91	5.22	0.25	0.49	<0.01	0.08
Starch-based protein/energy supplement DMI, kg/d ^6^	0.00	1.93	0.00	1.94	0.26	0.79	<0.01	0.81
Total DMI, kg/d	4.63	7.87	5.34	7.57	0.29	0.50	<0.01	0.11
Gain:Feed	0.078	0.105	0.059	0.118	0.009	0.72	< 0.01	0.10

^1^ NoVTM: No vitamin mineral supplement was a pelleted product fed at a 0.45 kg•heifer^−1^•day^−1^ with no added vitamin and mineral supplement. ^2^ VTM: Vitamin mineral supplement was a pelleted product fed at a 0.45 kg•heifer^−1^•day^−1^ (consisting of 113 g of a vitamin and mineral supplement, formulated to deliver similar levels of vitamins and minerals that were fed pre-breeding and 337 g of a carrier). ^3^ Heifers fed a pelleted blend of ground corn, dried distillers grains plus solubles, wheat midds, fish oil, urea, and ethoxyquin fed at rate to achieve targeted gain of 0.79 kg/d for MG treatment. ^4^ NoVTM-LG (*n* = 9); NoVTM-MG (*n* = 9); VTM-LG (*n* = 9); VTM-MG (*n* = 8). ^5^ Calculated from the time of artificial insemination to d 83 of gestation. ^6^ Arthimetic means presented for consumption of the protein/energy supplement.

**Table 5:** Concentrations of insulin and insulin-like growth factor 1 (IGF-1) in serum from beef heifers as influenced by vitamin and mineral supplementation [VMSUP; not supplemented (NoVTM) or supplemented (VTM)] and two different rates of gain [GAIN; low rate, 0.28 kg/d (LG) or moderate rate, 0.79 kg/d (MG)].

Item	NoVTM ^1^		VTM ^2^		SEM ^4^	*p*-Values						
	LG	MG ^3^	LG	MG ^3^		Day	VMSUP	GAIN	VMSUP × GAIN	VMSUP × Day	GAIN × Day	VMSUP × GAIN × Day
Insulin, pmol/L												
Prior to VMSUP ^5^	31.07	27.58	29.22	16.56	5.97	<0.01	0.01	0.16	0.36	0.30	0.09	0.74
Pre-breeding ^6^	46.47	36.09	34.00	20.61	5.97							
d 83 of gestation ^7^	54.92	62.86	45.55	40.94	5.97							
IGF-1, nmol/L												
Prior to VMSUP ^5^	17.51	20.91	19.24	19.24	1.38	<0.01	0.49	0.25	0.47	0.42	<0.01	0.30
Pre-breeding ^6^	19.17	18.95	21.36	19.06	1.38							
d 83 of gestation ^7^	12.42 ^b^	15.99 ^a^	13.83 ^b^	17.54 ^a^	1.38							

^1^ NoVTM: No vitamin and mineral supplement was a pelleted product fed at a 0.45 kg•heifer^−1^•day^−1^ with no added vitamin and mineral supplement. ^2^ VTM: Vitamin mineral supplement was a pelleted product fed at a 0.45 kg•heifer^−1^•day^−1^ (consisting of 113 g of a mineral and vitamin supplement, formulated to deliver similar levels of vitamins and minerals that were fed pre-breeding and 337 g of a carrier). ^3^ Heifers fed a pelleted blend of ground corn, dried distillers grains plus solubles, wheat midds, fish oil, urea, and ethoxyquin fed at rate to achieve targeted gain of 0.79 kg/d. ^4^ NoVTM-LG (*n* = 9); NoVTM-MG (*n* = 9); VTM-LG (*n* = 9); VTM-MG (*n* = 8). ^5^ Blood was collected before VMSUP factor initiation (71 to 148 d before artificial insemination). ^6^ Blood was collected pre-breeding (at the time of CIDR insertion—9 d prior to artificial insemination). ^7^ Blood was collected at the time of ovariohysterectomy (d 83 ± 0.27 after artificial insemination).

**Table 6:** Effect of feeding a vitamin and mineral supplement [VMSUP; not supplemented (NoVTM) or supplemented (VTM)] and two different rates of gain [GAIN; low rate, 0.28 kg/d (LG) or moderate rate, 0.79 kg/d (MG)] to beef heifers during the first trimester of gestation on dam reproductive tract characteristics at day 83 ± 0.27 of gestation.

Item	NoVTM ^1^		VTM ^2^			*p*-Values		
	LG	MG ^3^	LG	MG ^3^	SEM ^4^	VMSUP	GAIN	VMSUP × GAIN
Gravid Uterus, g	1,916.9	1,765.4	1,763.5	1,757.0	100.22	0.44	0.43	0.49
Empty Uterus. g	446.78	409.56	435.70	460.20	20.49	0.35	0.75	0.16
Corpus luteum, g	3.89	4.78	3.89	4.93	0.29	0.79	0.003	0.82
Corpus luteum diameter, cm	3.40	3.67	3.35	3.92	0.16	0.55	0.02	0.39
Largest placentomes ^5^, cm	3.27	3.44	3.76	3.73	0.29	0.21	0.81	0.75

^1^ NoVTM: No vitamin and mineral supplement was a pelleted product fed at a 0.45 kg•heifer^−1^•day^−1^ with no added vitamin and mineral supplement. ^2^ VTM: Vitamin mineral supplement was a pelleted product fed at a 0.45 kg•heifer^−1^•day^−1^ (consisting of 113 g of a vitamin and mineral supplement, formulated to deliver similar levels of vitamins and minerals that were fed pre-breeding and 337 g of a carrier). ^3^ Heifers fed a pelleted blend of ground corn, dried distillers grains plus solubles, wheat midds, fish oil, urea, and ethoxyquin fed at rate to achieve targeted gain of 0.79 kg/d. ^4^ NoVTM-LG (*n* = 9); NoVTM-MG (*n* = 9); VTM-LG (*n* = 9); VTM-MG (*n* = 8). ^5^ Mean diameter of 3 largest placentomes in the reproductive tract.

**Table 7:** Effect of feeding a vitamin and mineral supplement [VMSUP; not supplemented (NoVTM) or supplemented (VTM)] and two different rates of gain [GAIN; low rate, 0.28 kg/d (LG) or moderate rate, 0.79 kg/d (MG)] to beef heifers during the first trimester of gestation on fetal and fetal organ mass and fetal body measurements at d 83 ± 0.27 of gestation.

Item	NoVTM ^1^		VTM ^2^			*p*-Values		
	LG	MG ^3^	LG	MG ^3^	SEM ^4^	VMSUP	GAIN	VMSUP × GAIN
Fetal mass, g								
Body	117.46	117.24	116.16	125.78	4.26	0.41	0.27	0.28
Liver	4.50	4.34	4.70	4.90	0.18	0.05	0.90	0.33
Heart	1.02	1.00	1.10	1.07	0.06	0.27	0.66	0.89
Intestine	2.56 ^ab^	2.47 ^ab^	2.42 ^a^	2.87 ^b^	0.11	0.24	0.09	0.03
Pancreas	0.29	0.27	0.27	0.29	0.04	0.96	0.97	0.57
Hindlimb	7.71	7.70	7.51	7.52	0.46	0.69	0.99	0.98
Femur	0.34	0.36	0.35	0.42	0.02	0.08	0.01	0.22
Brain	3.67	3.51	3.60	3.67	0.18	0.84	0.79	0.56
Ovaries	0.06	0.07	0.07	0.06	0.01	0.81	0.74	0.16
Fetal mass, % of fetal BW								
Liver	3.85	3.72	4.07	3.85	0.08	0.05	0.04	0.62
Heart	0.86	0.86	0.97	0.86	0.04	0.16	0.20	0.19
Intestine	2.15	2.19	2.13	2.25	0.09	0.86	0.34	0.67
Pancreas	0.27	0.21	0.22	0.22	0.04	0.53	0.46	0.37
Hindlimb	6.59	6.52	6.39	5.93	0.27	0.14	0.33	0.46
Femur	0.26	0.32	0.3	0.33	0.02	0.19	0.07	0.42
Brain	3.15	2.95	3.08	2.91	0.15	0.70	0.22	0.90
Ovaries	0.05	0.06	0.06	0.05	0.01	0.68	0.46	0.08
Measurement, cm								
Crown rump length	14.36	14.71	14.41	14.57	0.213	0.83	0.24	0.67
Curved crown-rump length	15.49	15.95	15.85	15.74	0.281	0.81	0.53	0.34
Eye diameter	1.29	1.30	1.28	1.38	0.074	0.64	0.46	0.55
Nose to poll	4.95	4.87	4.96	5.07	0.075	0.19	0.85	0.23
Body depth at front shoulder	4.19	4.09	4.16	4.25	0.065	0.38	0.91	0.15
Body depth at umbilicus	3.59	3.53	3.43	3.69	0.077	0.99	0.20	0.06

^ab^ Means within row lacking common superscript differ (*p* ≤ 0.05). ^1^ NoVTM: No vitamin and mineral supplement was a pelleted product fed at a 0.45 kg•heifer^−1^•day^−1^ with no added vitamin and mineral supplement. ^2^ VTM: Vitamin mineral supplement was a pelleted product fed at a 0.45 kg•heifer^−1^•day^−1^ (consisting of 113 g of a vitamin and mineral supplement formulated to deliver similar levels of vitamins and minerals that were fed pre-breeding and 337 g of a carrier). ^3^ Heifers fed a pelleted blend of ground corn, dried distillers grains plus solubles, wheat midds, fish oil, urea, and ethoxyquin fed at rate to achieve targeted gain of 0.79 kg/d. ^4^ NoVTM-LG (*n* = 9); NoVTM-MG (*n* = 9); VTM-LG (*n* = 9); VTM-MG (*n* = 8).

## Data Availability

The data presented in this study are available on request from the corresponding author.

## References

[1] Caton J.S., Crouse M.S., Reynolds L.P., Neville T.L., Dahlen C.R., Ward A.K., Swanson K.C. (2019). Maternal nutrition and programming of offspring energy requirements. Transl. Anim. Sci..

[2] Crouse M.S., Greseth N.P., McLean K.J., Crosswhite M.R., Pereira N.N., Ward A.K., Reynolds L.P., Dahlen C.R., Neville B.W., Borowicz P.P. (2019). Maternal nutrition and stage of early pregnancy in beef heifers: Impacts on hexose and AA concentrations in maternal and fetal fluids. J. Anim. Sci..

[3] Caton J.S., Crouse M.S., McLean K.J., Dahlen C.R., Ward A.K., Cushman R.A., Grazul-Bilska A.T., Neville B.W., Borowicz P.P., Reynolds L.P. (2020). Maternal periconceptual nutrition, early pregnancy, and developmental outcomes in beef cattle. J. Anim. Sci..

[4] Wu G., Bazer F.W., Dai Z., Li D., Wang J., Wu Z. (2014). Amino acid nutrition in animals: Protein synthesis and beyond. Ann. Rev. Anim. Biosci..

[5] Reynolds L.P., Borowicz P.P., Caton J.S., Vonnahme K.A., Luther J.S., Hammer C.J., Maddock Carlin K.R., Grazul-Bilska A.T., Redmer D.A. (2010). Developmental programming: The concept, large animal models, and the key role of uteroplacental vascular development. J. Anim. Sci..

[6] Reynolds L.P., Borowicz P.P., Caton J.S., Crouse M.S., Dahlen C.R., Ward A.K. (2019). Developmental programming of fetal growth and development. Vet. Clin. N. Am. Food Anim. Pract..

[7] Price D.M., Arellano K.K., Irsik M., Rae D.O., Yelich J.v., Mjoun K., Hersom M.J. (2017). Effects of trace mineral supplement source during gestation and lactation in angus and brangus cows and subsequent calf immunoglobulin concentrations, growth, and development. Prof. Anim. Sci..

[8] Reynolds L.P., Vonnahme K.A. (2017). Livestock as models for developmental programming. Anim. Front..

[9] Crouse M.S., McLean K.J., Greseth N.P., Crosswhite M.R., Negrin Pereira N., Ward A.K., Reynolds L.P., Dahlen C.R., Neville B.W., Borowicz P.P. (2017). Maternal nutrition and stage of early pregnancy in beef heifers: Impacts on expression of glucose, fructose, and cationic amino acid transporters in utero-placental tissues. J. Anim. Sci..

[10] Crouse M.S., Caton J.S., Cushman R.A., McLean K.J., Dahlen C.R., Borowicz P.P., Reynolds L.P., Ward A.K. (2019). Moderate nutrient restriction of beef heifers alters expression of genes associated with tissue metabolism, accretion, and function in fetal liver, muscle, and cerebrum by day 50 of gestation. Transl. Anim. Sci..

[11] Crouse M.S., McLean K.J., Dwamena J., Neville T.L., Menezes A.C.B., Ward A.K., Reynolds L.P., Dahlen C.R., Neville B.W., Borowicz P.P. (2021). The effects of maternal nutrition during the first 50 d of gestation on the location and abundance of hexose and cationic amino acid transporters in beef heifer uteroplacental tissues. J. Anim. Sci..

[12] Davy J.S., Forero L.C., Shapero M.W.K., Rao D.R., Becchetti T.A., Koopman Rivers C., Stackhouse J.W., Deatley K.L., McNabb B.R. (2019). Mineral status of california beef cattle. Transl. Anim. Sci..

[13] Olson K.C. Cow supplementation: Getting the best bang for your buck. Proceedings of the Range Beef Cow Symposium XXIV.

[14] Olson K.C. Delivery of supplements on rangelands. Proceedings of the Range Beef Cow Symposium XX.

[15] Menezes A.C.B., McCarthy K.L., Kassetas C.J., Baumgaertner F., Kirsch J.D., Dorsam S., Neville T.L., Ward A.K., Borowicz P.P., Reynolds L.P. (2021). Vitamin and mineral supplementation and rate of gain during the first trimester of gestation affect concentrations of amino acids in maternal serum and allantoic fluid of beef heifers. J. Anim. Sci..

[16] Hay W.W. (2006). Placental-fetal glucose exchange and fetal glucose metabolism. Transact. Am. Clin. Climatol. Assoc..

[17] Ticiani E., Rodrigues V.H.V., Willhelm B.R., Ribeiro E., de Costa Gerger R.P., Miglino M.A., Ambrosio C., Ferrell C., Sainz R.D., Rodrigues J.L. (2020). Biochemical and metabolic profiles in in vivo- and in vitro-derived concepti in cattle. Livest. Sci..

[18] Diniz W.J.S., Reynolds L.P., Borowicz P.P., Ward A.K., Sedivec K.K., McCarthy K.L., Kassetas C.J., Baumgaertner F., Kirsch J.D., Dorsam S.T. (2021). Maternal vitamin and mineral supplementation and rate of maternal weight gain affects placental expression of energy metabolism and transport-related genes. Genes.

[19] National Academy of Sciences Engineering and Medicine (2016). Nutrient Requirements of Beef Cattle: Eighth Revised Edition.

[20] Lamb G.C., Dahlen C.R., Larson J.E., Marquezini G., Stevenson J.S. (2010). Control of the estrous cycle to improve fertility for fixed-time artificial insemination in beef cattle: A review. J. Anim. Sci..

[21] Lamb G.C., Dahlen C.R., Brown D.R. (2003). Reproductive ultrasonography for monitoring ovarian structure development, fetal development, embryo survival, and twins in beef cows. Prof. Anim. Sci..

[22] Goetsch A.L., Murphy G.E., Grant E.W., Forster L.A., Galloway D.L., West C.P., Johnson Z.B. (1991). Effects of animal and supplement characteristics on average daily gain of grazing beef caltle. J. Anim. Sci..

[23] Cappellozza B.I., Cooke R.F., Filho T.A.G., Bohnert D.W. (2014). Supplementation based on protein or energy ingredients to beef cattle consuming low-quality cool-season forages: I. Forage disappearance parameters in rumen-fistulated steers and physiological responses in pregnant Heifers 1. J. Anim. Sci..

[24] Cappellozza B.I., Cooke R.F., Reis M.M., Moriel P., Keisler D.H., Bohnert D.W. (2014). Supplementation based on protein or energy ingredients to beef cattle consuming low-quality cool-season forages: II. Performance, reproductive, and metabolic responses of replacement Heifers 1. J. Anim. Sci..

[25] Van Soest P.J., Robertson J.B., Lewis B.A. (1991). Methods for dietary fiber, neutral detergent fiber, and nonstarch polysaccharides in relation to animal nutrition. J. Dairy Sci..

[26] Rodrigues M.S., Cooke R.F., Marques R.S., Arispe S.A., Keisler D.H., Bohnert D.W. (2015). Effects of oral meloxicam administration to beef cattle receiving lipopolysaccharide administration or vaccination against respiratory challenges. J. Anim. Sci..

[27] Beckett L., Gleason C.B., Bedford A., Liebe D., Yohe T.T., Hall M.B., Daniels K.M., White R.R. (2021). Rumen volatile fatty acid molar proportions, rumen epithelial gene expression, and blood metabolite concentration responses to ruminally degradable starch and fiber supplies. J. Dairy Sci..

[28] Lippolis K.D., Cooke R.F., Schumaher T., Brandão A.P., Silva L.G.T., Schubach K.M., Marques R.S., Bohnert D.W. (2017). Physiologic, health, and performance responses of beef steers supplemented with an immunomodulatory feed ingredient during feedlot receiving. J. Anim. Sci..

[29] Leiva T., Cooke R.F., Brandão A.P., Schubach K.M., Batista L.F.D., Miranda M.F., Colombo E.A., Rodrigues R.O., Junior J.R.G., Cerri R.L.A. (2017). Supplementing an immunomodulatory feed ingredient to modulate thermoregulation, physiologic, and production responses in lactating dairy cows under heat stress conditions. J. Dairy Sci..

[30] Oosthuizen N., Fontes P.L.P., Henry D.D., Ciriaco F.M., Sanford C.D., Canal L.B., de Moraes G.v., Dilorenzo N., Currin J.F., Clark S. (2018). Administration of recombinant bovine somatotropin prior to fixed-time artificial insemination and the effects on fertility, embryo, and fetal size in beef heifers. J. Anim. Sci..

[31] McLean K.J., Dahlen C.R., Borowicz P.P., Reynolds L.P., Crosswhite M.R., Neville B.W., Walden S.D., Caton J.S. (2016). Technical note: A new surgical technique for ovariohysterectomy during early pregnancy in beef heifers. J. Anim. Sci..

[32] D’Occhio M.J., Baruselli P.S., Campanile G. (2019). Influence of nutrition, body condition, and metabolic status on reproduction in female beef cattle: A review. Theriogenology.

[33] Noya A., Casasús I., Rodríguez-Sánchez J.A., Ferrer J., Sanz A. (2020). A negative energy balance during the peri-implantational period reduces dam IGF-1 but does not alter progesterone or pregnancy-specific protein B (PSPB) or fertility in suckled cows. Domest. Anim. Endocrinol..

[34] Rocha C.C., Martins T., Cardoso B.O., Silva L.A., Binelli M., Pugliesi G. (2019). Ultrasonography-accessed luteal size endpoint that most closely associates with circulating progesterone during the estrous cycle and early pregnancy in beef cows. Anim. Reprod. Sci..

[35] Evans A.C.O., Mossa F., Walsh S.W., Scheetz D., Jimenez-Krassel F., Ireland J.L.H., Smith G.W., Ireland J.J. (2012). Effects of maternal environment during gestation on ovarian folliculogenesis and consequences for fertility in bovine offspring. Reprod. Domest. Anim..

[36] Mann G.E., Fray M.D., Lamming G.E. (2006). Effects of time of progesterone supplementation on embryo development and interferon-τ production in the cow. Vet. J..

[37] Lonergan P., Forde N., Spencer T. (2016). Role of progesterone in embryo development in cattle. Reprod. Fertil. Dev..

[38] Rodríguez-Sánchez J.A., Sanz A., Ferrer J., Casasús I. (2018). Influence of postweaning feeding management of beef heifers on performance and physiological profiles through rearing and first lactation. Domest. Anim. Endocrinol..

[39] Mense K., Heidekorn-Dettmer J., Wirthgen E., Brockelmann Y., Bortfeldt R., Peter S., Jung M., Höflich C., Hoeflich A., Schmicke M. (2018). Increased concentrations of insulin-like growth factor binding protein (IGFBP)-2, IGFBP-3, and IGFBP-4 are associated with fetal mortality in pregnant cows. Front. Endocrinol..

[40] Prezotto L.D., Lemley C.O., Camacho L.E., Doscher F.E., Meyer A.M., Caton J.S., Awda B.J., Vonnahme K.A., Swanson K.C. (2014). Effects of nutrient restriction and melatonin supplementation on maternal and foetal hepatic and small intestinal energy utilization. J. Anim. Physiol. Anim. Nutr..

[41] Prezotto L.D., Camacho L.E., Lemley C.O., Keomanivong F.E., Caton J.S., Vonnahme K.A., Swanson K.C. (2016). Nutrient restriction and realimentation in beef cows during early and mid-gestation and maternal and fetal hepatic and small intestinal in vitro oxygen consumption. Animal.

[42] Prezotto L.D., Thorson J.F., Borowicz P.P., Peine J.L., Bedenbaugh M., Hileman S.M., Lents C.A., Caton J.S., Swanson K.C. (2018). Influences of maternal nutrient restriction and arginine supplementation on visceral metabolism and hypothalamic circuitry of offspring. Domest. Anim. Endocrinol..

[43] Chen Y., Arsenault R., Napper S., Griebel P. (2015). Models and methods to investigate acute stress responses in cattle. Animals.

[44] Sternbauer K., Luthman J. (2002). Insulin sensitivity of heifers on different diets. Acta Vet. Scand..

[45] Deters E.L., Hansen S.L. (2020). INVITED REVIEW: Linking road transportation with oxidative stress in cattle and other species. Appl. Anim. Sci..

[46] Dubey P., Thakur V., Chattopadhyay M. (2020). Role of minerals and trace elements in diabetes and insulin resistance. Nutrients.

[47] Marriott B.P., Birt D., Stallings V., Yates A. (2020). Present Knowledge in Nutrition: Clinical and Applied Topics in Nutrition.

[48] Meyer A.M., Caton J.S. (2016). Role of the small intestine in developmental programming: Impact of maternal nutrition on the dam and offspring. Adv. Nutr..

